# Needs and Concerns Regarding a Pediatric Palliative Telehealth App for Use in Palliative Home Care for Adults Among Providers in Germany: Embedded Mixed Methods Study

**DOI:** 10.2196/92048

**Published:** 2026-07-31

**Authors:** Rebekka Hocher, Sandra Weyandt, Jannik Zimmermann, Sebastian Fischer, Katharina Seibel, Gerhild Becker, Michaela Nathrath, Thomas Voelker, Merlin Deckers

**Affiliations:** 1 Kleine Riesen Nordhessen gGmbH - Palliative Care Team for Children Kassel, Mönchebergstr. 41-43, Kassel, Hesse, 34125, Germany, 49 561-980-1755, 49 561-980-6819; 2 Department of Palliative Care, Medical Center and Faculty of Medicine, University of Freiburg, Freiburg, Baden-Wurttemberg, Germany; 3 Theory and Methodology of Counseling, Department of Psychology, University of Kassel, Kassel, Hesse, Germany; 4 PalliativTeam Frankfurt gemeinnützige GmbH, Frankfurt, Hesse, Germany; 5 Clinic for Pediatric Hemato-Oncology, Psychosomatics and Systemic Diseases, Klinikum Kassel, Kassel, Hesse, Germany; 6 Children's Hospital, Technical University of Munich, Munich, Bavaria, Germany

**Keywords:** telemedicine, artificial intelligence, AI, mixed methods, needs assessment, focus groups, palliative care, home care services

## Abstract

**Background:**

Specialized outpatient palliative care (SOPC) provides home-based care for terminally ill patients and is associated with improved quality of life and prolonged survival. Due to its decentralized structure and growing demand, SOPC is a key target for digital transformation. Telehealth, mobile health, and AI offer considerable potential benefits but also present challenges, particularly with regard to user acceptance. Although both children and adults receive SOPC under the German statutory health insurance system, services differ in terms of patient characteristics, duration of care, and geographic coverage. Moreover, there is limited knowledge regarding the extent to which digital health applications are transferable across different areas of palliative care.

**Objective:**

This study assesses the extent to which needs and concerns regarding digitalization in SOPC for children are transferable to adult SOPC, using the PalliDoc Mobile app as a case example.

**Methods:**

Two adult SOPC teams using the PalliDoc Mobile app (a pediatric-origin mobile app) were surveyed between March 2022 and March 2025 using an embedded mixed methods design to assess digitalization needs and concerns. Therefore, a focus group study took place in the respective offices of the included teams. Twenty-five members from both teams, who were recruited via the personal network of the authors, participated, representing urban and rural care areas in Germany. Using an open-ended interview guide, the needs and concerns were first discussed with all participating members of each team. In a second step, the participants were allocated to profession-specific subgroups to further explore and prioritize the identified needs using a quantitative voting format. The focus group discussions were analyzed using qualitative content analysis, while the analysis of the prioritization votes was performed using descriptive statistics.

**Results:**

The triangulation of qualitative and quantitative findings revealed a total of 13 needs within the examined care teams for adults, with functions focusing on voice control being the highest priority (n=8 positive votes and no negative votes for voice input; n=6 positive votes and no negative votes for voice output). Additionally, functions relating to digitization and organizational tasks were viewed as predominantly helpful. Unlike in pediatrics, telehealth functions like video contacts, telemetry, and electronic patient-reported outcome measures are neither used here now nor intended to be used in the future. The identified concerns predominantly addressed the potential risk of AI-assisted documentation (n=2 positive votes and n=7 negative votes) altering or distorting health care professionals’ perception of information related to patients.

**Conclusions:**

Cross-setting telehealth applications may work, but they are no “plug-and-play solution.” Needs and concerns in each setting should be addressed to guarantee customized services.

## Introduction

Specialized outpatient palliative care (SOPC) provides home-based care for individuals who are severely ill and dying and has been associated with prolonged survival and improved quality of life [1]. One of the main challenges is the decentralized delivery of care across geographical distances, making SOPC a key target for digitalization efforts [2,3]. Digital transformation represents the broadest concept of digitalization, encompassing the pervasive integration of digital technologies and the reorganization of services [4]. Key components of the digital transformation of health care can be described using the overlapping terms digitization, digitalization, mobile health (mHealth), telehealth, and AI [4-6]. In the context of public health, digitization refers to the technical process of converting analog records into digital data, whereas digitalization refers to the integration of digital technologies into public health operations [4]. mHealth refers to the use of apps on mobile devices and overlaps with telehealth, which enables digital communication between patients receiving care and health care providers [4,5]. Telehealth encompasses the umbrella terms Telemedicine and Telecare and best describes the multidisciplinary delivery of palliative care [4]. Even though AI is becoming increasingly integrated into digitalization processes with the emergence of large language models [7,8], ongoing work suggests that health care professionals (HCPs) in palliative care do not use AI in their daily work regularly (68.77 %) [9], which raises questions about the acceptance of its integration.

The German care structure distinguishes between SOPC teams for adults and SOPC teams for children, adolescents, and young adults [10]. Pediatric SOPC is characterized by the involvement of the entire family system, a larger geographic care area, and longer care durations, with a comparatively lower focus on end-of-life support [10]. Additionally, home visits are often conducted jointly by a nurse and a physician [11]. In pediatric SOPC, most patients are individuals affected by rare diseases, primarily neuromuscular and neurodegenerative disorders (39%), followed by cancer (28%), chromosomal aberrations (8%), and cardiovascular conditions (8%) [11,12]. In contrast, patients receiving care from SOPC teams for adults predominantly present with cancer (55%), heart failure (16%), and dementia (8%) [13].

For pediatric SOPC, one of the first commercially available German-language apps, the PalliDoc Mobile app for Android and iOS (hereinafter referred to as “the app”), which facilitates digitization, digitalization, mHealth, and telehealth, is now available [14-16]. The app has been further developed through a participatory approach for pediatric SOPC [15] and includes a version for SOPC team members and a version for patients, both of which sync with each other and with the desktop version of the PalliDoc palliative patient documentation system [14,15]. While teams working with adults are using it, its suitability for this context remains unclear.

The creation of specific apps involves a significant amount of effort in terms of needs assessment and development and thus entails high costs [15-17]. For related health care sectors, therefore, adopting, and if necessary, adapting existing apps can be effective. However, transferability must be definitively determined through an evaluation in the target context to address potential barriers to implementation [18-20]. Beyond technical issues like data protection, acceptance by users plays a crucial role and largely depends on performance and effort expectancy [21-23].

Previous research has focused on general perceptions of telehealth in SOPC, but user-centered approaches remain crucial to ensure acceptance and practical value [23-30]. Therefore, this study examined the adaptation of the pediatric app for use in specialized outpatient palliative care for adults. Our aim is to assess the necessity of a customized solution for SOPC for adults and to identify practical paths for sustainable digital transformation needs by exploring the following research questions:

1. What digital transformation needs and concerns do professionals in the field of SOPC for adults have with regard to the pediatric app being used?
  In addition, the following subquestions were examined:
2. Which of the identified needs are prioritized by the team members in terms of their relevance?
3. What specific areas of application can be derived for the day-to-day practice of SOPC, and implemented through the further development of the existing app?

## Methods

In drafting the manuscript, we followed the 32-item COREQ (Consolidated Criteria for Reporting Qualitative Research) guidelines [31] to ensure transparency in all aspects of our embedded mixed methods study.

### Study Design

**Figure 1.**
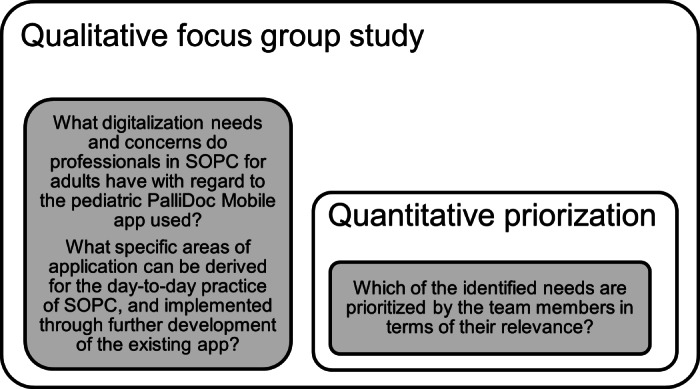
Embedded mixed methods design including a qualitative focus group study and quantitative prioritization, with its aligned research questions. SOPC: specialized outpatient palliative care.

As this is a new and still emerging research field, we opted for an embedded mixed methods design [32] to formatively evaluate the use of the pediatric app in the context of SOPC for adults. We, therefore, decided to conduct face-to-face discussions with SOPC teams for adults in the form of focus groups to effectively assess the individual needs and concerns of each SOPC team. To further assess which features should be prioritized in the further development of the app for adult SOPC, we carried out a quantitative needs assessment, which was incorporated as part of the focus groups (Figure 1). This approach allowed us to openly explore the assessment of digital transformation needs and concerns while simultaneously quantitatively mapping the needs considered to be particularly relevant by the participating SOPC team members.

### Setting

**Figure 2.**
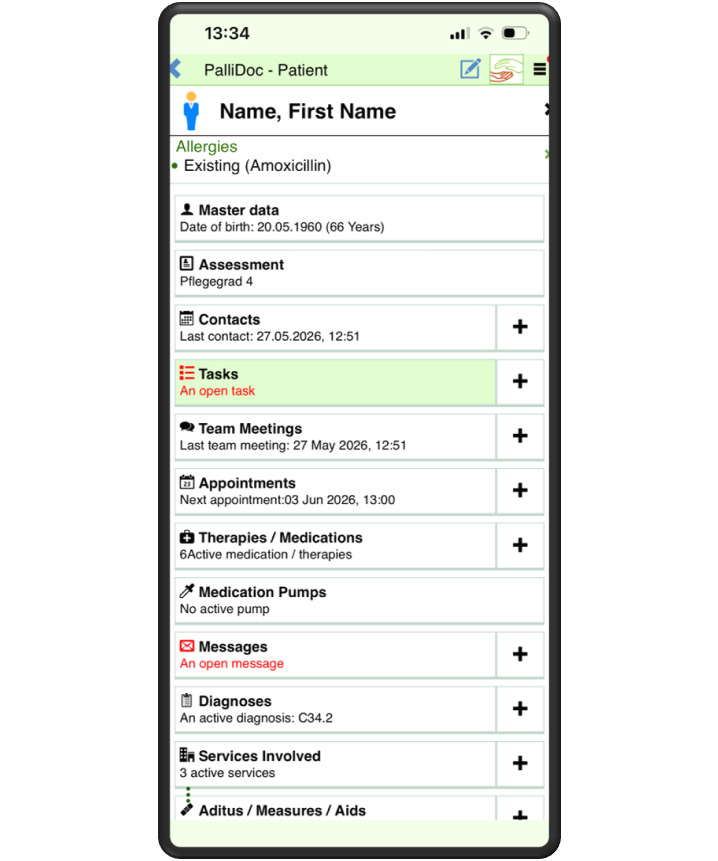
Screenshot of a section of the PalliDoc Mobile app home screen used by the adult palliative care teams participating in this study. “Messages” enables communication among health care professionals within the palliative care team*.* Communication between patients and providers was not used, as telehealth functionality had not been implemented. Pflegegrad 4: German for “level 4 for long-term care insurance”.

This study is part of the research project “TAPE” (Telehealth in Outpatient Palliative Care for Adults) and builds upon the previous project “TelPa_kids,” which developed the apps for patients and SOPC team members. These applications seamlessly synchronize with the PalliDoc patient documentation system for use in SOPC [14,16]. App development was based on a needs assessment conducted with patients, caregivers, and pediatric SOPC team members [15]. The patient app allows patients to access educational and informational materials, the electronic palliative patient file (ie, medication plan), upload data (documents, photos, and videos), document questionnaires, and electronic patient-reported outcomes (ePRO), as well as engage in live video contact [14-16]. The app for SOPC team members enables all processes required for the electronic data processing of patient care, such as documenting contacts, updating medication, creating prescriptions, and recording other entries in the electronic palliative patient file. In addition, it allows users to easily access contact information on their smartphones to make calls, compose emails, and navigate to the patient’s location. The app does not support administrative functions relevant to billing or care data analysis. When the patient app is made available, the range of features for SOPC team members’ apps will be expanded to include telehealth functions such as questionnaire assessment, ePRO, and video contact. Figure 2 provides an example of a screenshot showing some available functions for the SOPC teams.

The company distributing the app was once again available for this project and handled the technical implementation and deployment of the software. However, the prior and current research presented here were conducted independently of the provider’s subsequent technical implementation.

The TAPE project team is located in the federal state of Hesse, Germany, and is part of the Palliative Care Team for Children in Kassel, Germany. Since 5 members of the research team (MD, MN, TV, SW, and SF) serve in dual roles as researchers and practitioners in pediatric SOPC, they had unique insights into and access to the field of study, which led to a strong motivation to improve palliative home care through digitalization, but may also have influenced their perception and analysis of the data. The project team proceeded on the assumption that the pediatric App would also be suitable for adult care settings, but that there might be differences in terms of the scope of functions and areas of application.

### Sample and Recruitment

The sampling strategy used a combination of purposive sampling and convenience sampling. The aim of the purposive sampling for the focus group study was to select 2 typical cases of German SOPC teams for adults, representing 1 team with an urban service area and 1 team with a rural service area [33]. In addition, representatives of all professional groups involved in SOPC were to be recruited within the 2 teams in order to represent a broad spectrum of needs and concerns, including conflicting ones. The representatives had to already have experience with the primary app and be working in direct contact with patients, either physical or remote (eg, office clerks with contact via email, phone, or the app). The specific inclusion criteria were as follows: staff working in specialized outpatient palliative care for adults (eg, physicians, nurses, office clerks, social workers, and pastoral care workers), fluency in written and spoken German, staff with direct patient contact, and use of the PalliDoc Mobile app.

Team members who did not meet all of these criteria were excluded from the study. The total study duration was from March 2022 to March 2025 and primarily focused on the acquisition of participants, as well as the conduction and analysis of the focus groups. The recruitment of the 2 teams took place via the professional network of the authors. Initial contact with the rural team occurred between MD and the team leader in March 2022, as this team was interested in using the original children’s app for adult care and adapting it as part of a study. Due to their availability and willingness to participate, this team was the first to be included. Later, in October 2023, a member of the research team (MD) contacted the urban team by phone with the specific aim of recruiting a more diverse sample for the study. This researcher knew from previous personal contacts that the urban team also shared an interest in implementing the app and therefore acted as a gatekeeper for recruitment. It was communicated that, if possible, all team members from all professional groups should be present. However, participation in the focus groups was voluntary, so the final composition of the sample was random and not determined until the day of the session. The participants were informed that the discussion would focus on digital transformation needs and concerns (using the general German term “Digitalisierung,” English “digitalization”) regarding the use of the pediatric app. Due to the participants’ high level of intrinsic motivation and interest in further digitalizing their work, there was a very high level of participation and no objections to participating in the study. Once the urban team had been included in the sample, the recruitment was completed.

### Data Collection

#### Qualitative Focus Group Study

Focus groups were chosen as an interactive format to examine the teams’ interpretations and attitudes toward the research topic. To this end, an interview guide was adapted that had been developed and piloted as part of a previous study on needs and concerns related to digitalization in the field of pediatric SOPC; however, it was not piloted again (for detailed information on the development process of the interview guide [15]). To address potential power imbalances and group dynamics among the various professional groups [34], needs and possible concerns regarding digitalization were first discussed in the joint group and finally specified during subsequent profession-specific groups (medical, nursing, psychosocial, and administrative staff).

The focus groups took place in person at the respective offices of the included SOPC teams for adults between May 2024 and June 2024. Each session lasted about 120 minutes and was facilitated by 4 members of the research team from various professional backgrounds: a female nurse and palliative care master’s student (SW), a male nurse and health scientist (SF), a female health scientist (RH), and a male physician (MD). Apart from the researchers mentioned, no other individuals who were not participating in the focus group were present. In addition to facilitating subgroup moderation based on similar professional backgrounds, the interprofessional team enabled a multiprofessional perspective on the focus group content and complemented the varying levels of methodological expertise. While MD and RH were experienced in leading focus groups, SF and SW were moderating for the first time. The moderation team pursued the scientific goal of influencing the discussion as little as possible and formulating the key questions openly. If necessary, topics were explored in greater depth by asking follow-up questions until data saturation was reached or narrowed down if they strayed from the main subject.

As usual for focus groups, the interview began with a stimulus question to help all participants engage with the topic. In the main portion, moderated by RH and MD, 2 thematic blocks were addressed: the needs (A) and the concerns (B) regarding digitalization in SOPC for adults. The results were simultaneously recorded by SW and SF during the discussion, noted on moderation cards, and displayed on a flipchart for the participants at the conclusion of the focus group.

Following these thematic blocks (A and B), the participants were subsequently divided into profession-specific groups to identify profession-specific needs and concerns that may not have been sufficiently addressed in the overall group. In doing so, digitalization needs and concerns were addressed again. MD and RH moderated the physician-specific group while SW and SF moderated the nurse-specific group. The complete guideline is presented in Textbox 1. Social and pastoral workers were included in the nurse-specific group, and office clerks could choose which group they wanted to join. The results from the group discussions were also noted simultaneously by the moderators and finally summarized orally for the participants at the end of each profession-specific session.

Textbox 1. **Guideline for the qualitative focus group study, including two open-ended questions each for (1) the joint focus group and (2) profession-specific focus groups.**

**Joint focus group**

- Needs of the focus group with regard to a digital app
- Key question: What are typical problems in your daily work that digitalization could help with?
- Concerns of the focus group with regard to a digital app
- Key question: What are your concerns about digitalization?

**Profession-specific focus group**

- Additional needs and concerns
- Key question: What needs and concerns for your professional group have been lost in the group discussion?
- “Think aloud” for prioritization
- Key question: What went through your mind when you stuck the green and red dots?

#### Quantitative Prioritization

During the profession-specific discussions, the participants were asked to evaluate all generated needs in terms of their relevance. The profession-specific group sessions were therefore held in 2 separate rooms, each of which had a flipchart displaying the documented results of the overall focus group. To prioritize, the participants were each given 2 green and 2 red adhesive dots. The green dots had to be awarded for a need that was considered a priority and particularly important, while red dots could be awarded for needs that were associated with concerns or fears. The alignment of both green dots was required, whereas the assignment of red dots was not mandatory. In consultation with the participants, the vote was conducted in an open form: The participants approached the flip chart either individually or in small groups and assigned their points. Afterward, using a “think aloud” stimulation technique (Textbox 1), the participants were asked to share their thoughts and feelings during the scoring process.

Both the joint and profession-specific focus group sessions were audio-recorded and informed the subsequent analysis of the content.

### Data Analysis

#### Qualitative Focus Group Study

The data analysis was conducted between May 2024 and September 2024, following the content-structuring content analysis method described by Kuckartz et al [35]. First, the audio recording of the first focus group interview with the rural SOPC team was transcribed verbatim and pseudonymized. After familiarizing themselves with this initial transcript, a case summary in memo format was created, which summarized not only the needs and concerns but also the background, such as local and spatial conditions, as well as the context and structure of the focus group. In addition, anomalies and peculiarities were documented in the form of a reflection. For the second, urban focus group, the audio material was listened to in small segments; during this process, a pseudonymized case summary memo was created, in which statements regarding digitalization needs and concerns were paraphrased and summarized. The framework conditions and background information were also recorded at the beginning of this memo.

The qualitative data were analyzed using a consensus-based approach. Four members of the research team (SW, RH, MD, and SF) independently reviewed the transcript and collaboratively developed a category system in which the main categories were defined as “needs” and “concerns,” in accordance with the research question and interview guidelines. The subcategories were derived entirely inductively from the data (the complete category system can be found in Multimedia Appendices 1 and 2). A coding guideline developed from this served as the coding tool. All 4 researchers (SW, RH, MD, and SF) then jointly coded the entire material through an iterative process of discussion and refinement. They analyzed the categories for connections and differences and finally formulated abstractions—supplemented by meaningful verbatim quotes for documentation purposes. It was important that the meaning of the statements be preserved despite the abstraction process. In cases of uncertainty regarding the coding or interpretation of the results, ongoing discussions took place during coding meetings within the research team (SW, RH, MD, and SF) to ensure openness and reflexivity during the data analysis. Consensus was reached through discussion and negotiation of meaning, with particular attention given to maintaining coherence within and across categories.

Prior to the final written documentation of the results, a member check was conducted in July 2024 as an interim step. Participants from the respective SOPC teams were invited to attend a digital presentation and discussion of the preliminary results regarding the identified needs and their potential implementation. The objectives of this member check were to ensure transparency and to review the results for completeness and potential misunderstandings. Through two targeted questions, ambiguities were identified and clarified:

1. How important is it to you to be able to access the app using voice commands while in the car?
2. Do you agree that voice control is an essential requirement for this use case?

In this way, 3 categories of needs were refined. The member check was also documented in a memo based on an audio recording and was ultimately incorporated into the final results.

#### Quantitative Prioritization

The analysis of the prioritization of the needs, which emerged during the joint focus group discussion, took place based on a previously defined baseline that served as a reference point for assessing relevance. The assignment of a green dot was given a positive value of +1, and the assignment of a red dot was given a negative value of –1. In this way, the degree of priority and the existence of concerns could be quantified. The data collected from the prioritization process were descriptively analyzed by JZ, RH, and MD using IBM SPSS (version 26.0).

### Ethical Considerations

The study was approved by the Ethics Committee of the University of Kassel (05/13/24, EKFB01 No. 202416) and was conducted in accordance with the Declaration of Helsinki. All participants were comprehensively informed of their rights, and a written declaration of consent was obtained prior to the questionnaire survey and focus groups. All data were collected in pseudonymized form—each participant had been allocated a unique code, known only to the research team. For analysis, only the pseudonymized transcripts were used. The participants did not receive any form of compensation for their participation in the study.

## Results

### Qualitative Focus Groups Study

A total of 25 members from the 2 included SOPC teams for adults participated in the 2 focus groups (Table 1). This enabled the perspectives of 13 nurses, 8 physicians, 2 psychosocial workers, and 2 office clerks to be incorporated into the study. All participants, except for 1 office clerk in the rural team, had completed additional training in PC. There were no refusals or dropouts during the study.

The analysis of the focus groups of the investigated SOPC teams for adults showed that the needs and concerns regarding the further digitalization of their work and use of the app essentially address 3 functional groups. On the one hand, additional features were requested that have so far played no or only a subordinate role in child care. These features focused on voice control as well as digitization and organization. On the other hand, features with a focus on telehealth were hardly or not at all used by the teams for adults, although they are frequently used in child care [26].

**Table 1.** Sampling structure based on demographic characteristics and occupational groups.

Focus group	Rural	Urban
Total (female: male), n	13 (11:2)	12 (10:2)
Age (y)	50.6 (SD 13.4)	45.5 (SD 9.8)
Professional experience (y)	30.4 (SD 11.7)	21.7 (SD 12.0)
Professional experience in palliative care (y)	16.7 (SD 8.2)	5.7 (SD 4.0)
Physicians (n)	4	4
Nurses (n)	7	6
Social and/or pastoral workers (n)	—^a^	2
Office clerks (n)	2	—^a^

a Not applicable.

### Requested Features With a Focus on Digitalization

#### Voice Control

Participants of the included SOPC teams for adults consider the implementation of a function allowing voice input and voice output of content with voice control to be the most relevant. The desire for efficient use of long travel times especially becomes apparent in the rural area as their regional character includes a large supply area with long distances (average rural team: 250,000 km/y vs 67,194 km/y for the urban team, according to self-declaration):

*[…] It needs a system that learns from the language […] And for us quirky folks, this is something where the dialect (.) should also be more easily adopted into written German without mistakes.*

[Focus group, rural team, nurse]

However, the discussion also highlights concerns about privacy, road safety when using voice input while driving, and the length and accuracy of texts generated by voice input. There is strong controversy over how much AI should handle processing, summarizing, and editing language. On the one hand, AI is seen as an “all-round talent” and versatile tool for data collection and automated documentation. On the other hand, there is great mistrust in the generated content and concerns about the loss of the “human factor” and the “ability to think for oneself,” as it could lead to a decline in professional competence and even the displacement of jobs due to automation:

*[…] I would never use this microphone in my life. I don't. […] That is nothing to me. I write my own stuff and then I know what it says and I don't have to check it again […]*

[Focus group, urban team, physician]

#### Digitization and Organization

An additional need for digital tools to support organizational and administrative processes within teams for adults becomes apparent, including: (1) the introduction of electronic prescriptions, (2) the use of digital signatures (ie, Health Care Treatment Agreement with the SOPC team), (3) the option to import medication plans into the electronic health record, and (4) a notification function to be informed about new or urgent information in the system via push messages:

*[…] my wish would still be, which makes sense, if the dosage is included, that you get a pop-up right away (.). […] That there is an indicator for me, so to speak, "Ah, now this has to be reordered." That there wouldn't be a bottleneck for the patient at all.*

[Focus group, rural team, nurse]

Moreover, in an urban setting, there is an additional need for access to the national electronic health record, an automated completeness check of documentation, and digital route planning in order to optimize home visits logistically and economically. In the rural area, the focus lies on efficiently using the app for documentation while driving. Concerns include the practicality of certain functions, legal compliance, data security, and privacy protection when handling sensitive patient data:

*[…] And then when I document, I think: “Hm, now everyone can see everything, but maybe the patient doesn't want the statutory health insurance to get this or that information as well or who’s business is it?” […].*

[Focus group, urban team, physician]

### Requested Features With a Focus on Telehealth

The integration of telehealth was discussed more controversially in both SOPC teams for adults. Across regions and professions, digitalization is seen merely as a supplement to personal care and is restricted to interprofessional communication, for example, between physicians and nurses. In contrast to pediatric care, where patients and families receive the patient app to allow video contacts and ePRO assessment, the communication options in SOPC for adults are limited and restricted to the use of other means of communication, such as telephone, and where applicable, common messenger services. The latter are mainly used in urban practice in the psychosocial field and are critically assessed there, especially with regard to the lack of demarcation:

*[…] but then it got so out of hand, um, that I, um just (…) received a text message almost 24 hours a day, um, sometimes even in the middle of the night […]. For me it was difficult to re-establish that boundary […]*

[Focus group, urban team, social worker]

Even though telecommunication applications are considered to have the potential to reduce workload, there are major reservations regarding the technical requirements, the potential loss of personal contact with patients, and the possible overburdening of patients, their caregivers, and nursing staff:

*I'm afraid that, um, this might mean that home visits by doctors may take place less often (speaks quietly) or that I'll have more responsibility […] To be honest, I see it (..) not as my job as a nurse. I'll stick to that.*

[Focus group, rural team, nurse]

### Quantitative Prioritization

**Figure 3.**
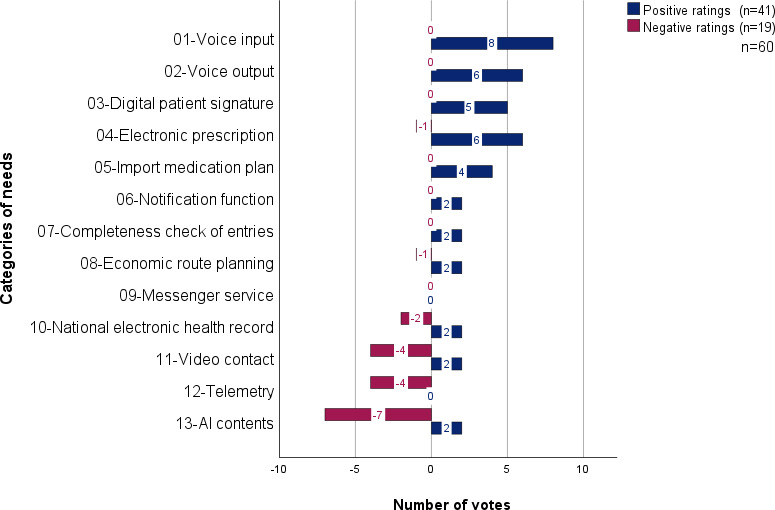
Results of the quantitative needs prioritization of identified categories of needs during the focus groups. Identification of further digitalization needs was conducted by 25 employees from 2 specialized outpatient palliative care (SOPC) teams for adults regarding priority functions (2 possible votes per employee) for the PalliDoc Mobile app along with the assignment of critical votes (0‐2 possible votes per employee). The SOPC teams surveyed use an electronic patient documentation system for SOPC. Germany implemented a national electronic health record in 2025 to facilitate data sharing between services. None of the desired features were already available to the adult SOPC teams. While the technical infrastructure for video calls with patients was in place, the patient app had not yet been made available to patients for this purpose.

The preference for digital functions that do not include telehealth components was also reflected in the analysis of the positive and negative rankings of the needs mentioned during the focus group discussion (Figure 3). A total of 60 votes were awarded, of which 41 were positive and 19 were negative. The integration of voice control in the app system was seen as most promising (n=8 positive and no negative votes for voice input; n=6 positive and no negative votes for voice output), whereas AI-powered content processing within the app was seen as the most critical aspect (n=2 positive and n=7 negative votes). As was already evident from the interviews, the integration of telehealth also received predominantly negative ratings (no positive and n=4 negative votes for telemetry; n=2 positive and n=4 negative votes for video contacts; no positive or negative votes for messenger services). Functions addressing digitization or organizational tasks were viewed as mostly positive (n=5 positive and no negative votes for digital patient signature; n=6 positive and n=1 negative votes for electronic prescription; n=4 positive and no negative votes for import of medication plan; n=2 positive and no negative votes for notification function and completeness check of entries; n=2 positive and n=1 negative votes for economic route planning).

### Areas of Application for the Day-to-Day Practice of SOPC for Adults

**Figure 4.**
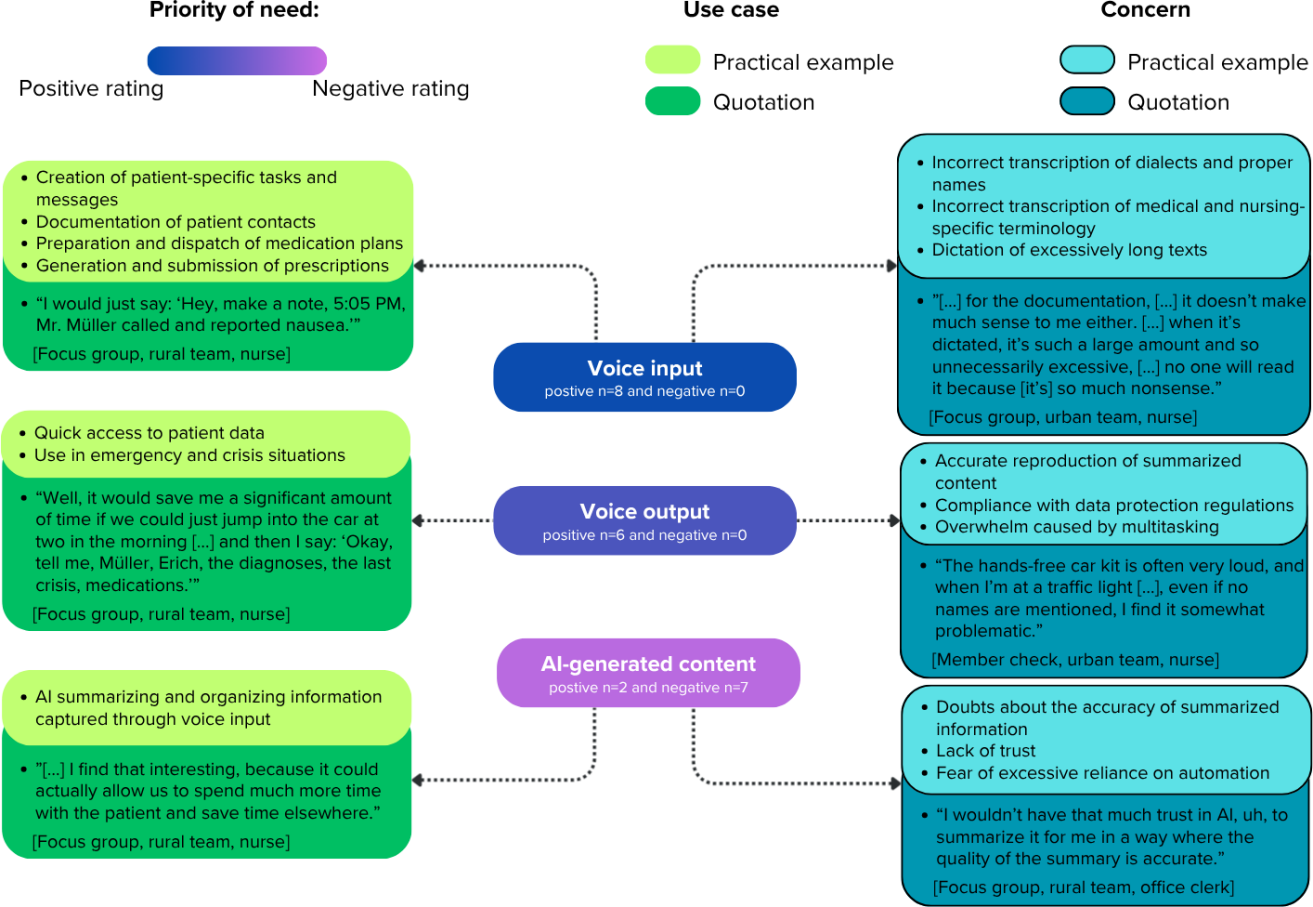
Joint display of digitalization and AI applications and barriers in specialized outpatient palliative care (SOPC) practice for adults. Quantitative data: color coding reflects the priority ratings of needs by staff (adult care). Qualitative data: practical examples illustrate subcategories of identified needs, along with representative quotations.

**Figure 5.**
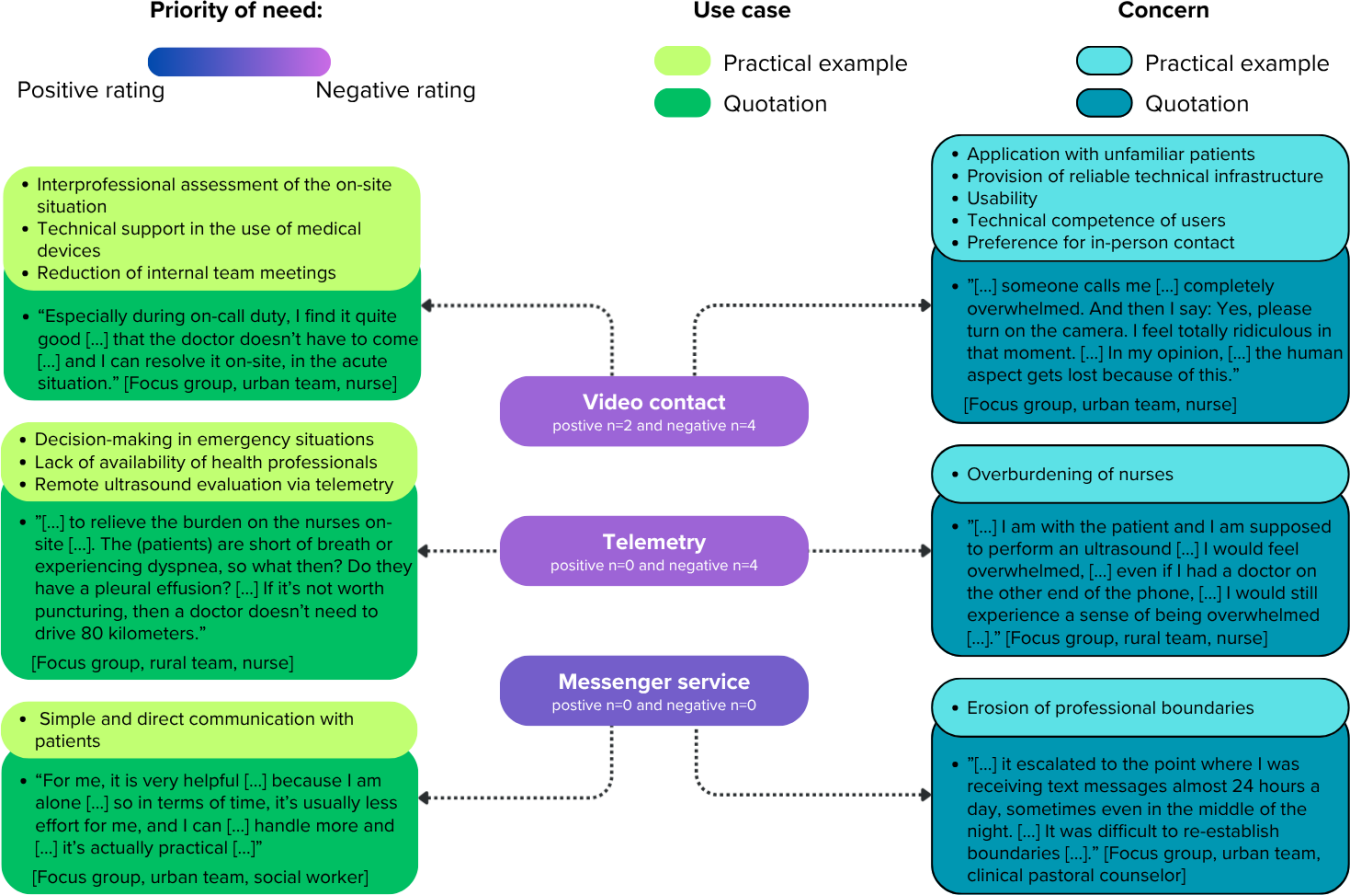
Joint display of telehealth applications and barriers in Specialized outpatient palliative care (SOPC) practice for adults. Quantitative data: color coding reflects the priority ratings of needs by staff (adult care). Qualitative data: practical examples illustrate subcategories of identified needs, along with representative quotations.

To derive concrete use cases for practical application in the field of SOPC, we further supplemented the qualitative data with the quantitative results from the needs prioritization in a joint display. To this end, 3 members of the research team (SF, SW, and MD) reviewed the material for clues and points of reference drawn from specific everyday situations and used them to develop possible scenarios for clinical practice. We therefore merged the identified categories of needs along with their frequency of positive and negative ratings and a key quote, which represents (1) one central field of application in clinical practice and (2) a major concern referring to this category. For better readability, the practical use cases were divided following the respective main categories: digitalization, “features with focus on voice control” (Figure 4), and telehealth, “features with focus on telehealth” (Figure 5).

## Discussion

### Main Results

In this study, we examined the key needs and concerns of SOPC staff for adults when transferring a digital application from the pediatric palliative care sector to the adult sector. We found that SOPC teams for adults seem to be satisfied with the existing app. However, both qualitative and quantitative findings show that telehealth, which was prioritized by pediatric SOPC teams [15], is neither currently used nor desired by teams providing care for adults. Instead, they requested a wide range of digitization and digitalization features that go beyond the current functional range of the existing app. This includes above all features like voice-controlled input and output for the patient documentation system, enabling a single HCP to efficiently prepare for and document home visits while driving. Despite acknowledging this potential, SOPC staff for adults remain reluctant to trust AI-generated summaries and AI-edited voice input in documentation, illustrating a form of algorithm aversion [30,36-38]. Additionally, they expressed a strong need for features that support organizational and administrative tasks, such as enabling digital signatures and streamlining the introduction of e-prescriptions.

### Implications

Digitalization in SOPC appears to be a complex and sensitive process. The needs and concerns regarding digitalization measures, such as the integration of apps, can vary significantly even within a single care setting—such as the SOPC—depending on the patient group or geographic area. Nevertheless, HCPs are satisfied with the support provided by mHealth. However, to realize the full potential of digitalization measures, specific, tailored solutions are needed, particularly in light of the growing need for palliative care against the backdrop of staff shortages. The mixed method approach presented here, along with the materials provided in the supplementary material including the category system for the identified needs (Multimedia Appendix 1) and concerns (Multimedia Appendix 2); all freely available under a Creative Commons License 4.0), can serve as a template for implementing, evaluating, and adapting technical solutions from related care sectors to enable rapid, effective, and cost-efficient digitalization [1].

To evaluate the user experience in digitalization, the fundamental concept of the Unified Theory of Acceptance and Use of Technology model [38] can be used here. This allows for the classification of user acceptance based on two underlying factors that depend directly on the technology used: (1) perceived usefulness, which is the main factor mediating perceived performance; and (2) usability, which serves as the main factor mediating effort expectancy. To measure these 2 factors, the 6-item Telehealth Usability and Perceived Usefulness Short Questionnaire is available, which is based on a longer questionnaire and available in many languages [39-42]. This allows the user experience to be evaluated and classified after an app has been implemented.

In this specific pilot project, for example, the low acceptance of telehealth and expressed reservations toward video contacts and AI in SOPC for adults can be attributed to the following factors: (1) perceived usefulness is lower in health care due to significantly shorter distances and a higher proportion of end-of-life care for individuals who are dying, where telehealth is clearly not an option, and (2) effort expectancy is higher, with an assumed lower affinity for technology among significantly older patients and their relatives. This assessment is consistent with surveys on technology acceptance and age [22,39,43]. When introducing AI, algorithm-based documentation has the potential to change the perception of individual patient care among HCPs and must be made comprehensible to users to avoid distortion and prevent misinformation.

Furthermore, our results show that HCPs with no experience in telehealth and the regular use of video contacts critically assess its potential use in SOPC for adults and do not use this function. Lundereng et al [30] reported 18 pilot studies that used video contact in SOPC and summarized mixed assessments from HCPs regarding its feasibility and usefulness. It remains unclear to what extent the piloting of video contacts was desired by HCPs or policymakers and whether its use could be continued after the pilot studies were completed. In addition, no HCPs in our study advocated the use of telehealth for monitoring in SOPC, as described in 16 pilot studies by Lundereng et al [30], and individual studies show challenges in maintaining its use [44,45]. Our ongoing evaluation of the TelPa_kids project also shows low and unsustained use of video contacts in pediatric SOPC [16]. This suggests that the continued use of telehealth in the daily practice of SOPC seems to be challenging beyond its use in pilot projects, as described for nursing homes, for example [45]. Nevertheless, differences in HCPs' attitudes and urban vs rural care settings offer valuable insights for international contexts, as supraregional findings from all over the globe indicate that telehealth may improve access to palliative care, especially in structurally weak and geographically dispersed care areas and thus save time and resources [46-50].

### Strengths and Limitations

This study is the first to explicitly investigate concrete digitalization needs and concerns in SOPC for adults, particularly with regard to the use of telehealth as implemented via a digital application adapted from the pediatric care context. However, differences in care models and geographical distances restrict the extent to which the German context can be applied to international SOPC. The study assessed further needs and concerns based on the use of a specific app (the PalliDoc Mobile app) [14], which also limits the transferability of the results. Moreover, the small number of only 2 SOPC teams with a total of 25 participants in the focus groups limits the generalizability of the findings. However, this comparative analysis with 2 specific teams enables a detailed examination of team-specific differences in digital transformation and may provide a roadmap for individual teams seeking to advance their digital transformation efforts by addressing their specific needs and concerns. Therefore, our mixed methods approach allows for the rapid assessment of digitalization measures. Methodologically, the prioritization technique using open votes could have led to biased results. Also, the reliability of focus groups is influenced by social dynamics, and preexisting team consensus may have overshadowed individual opinions on additional issues [51].

### Conclusions

The provision of SOPC for patients who are severely ill and dying in their homes is a delicate and individualized process that depends on patient characteristics and the geographic area covered. It is associated with distinct team-specific challenges and corresponding needs and concerns related to digitalization. Based on our findings, customized solutions seem to be needed in order to achieve a sustainable improvement in the use of new technologies in SOPC, as cross-setting solutions may lead to a certain degree of satisfaction but ultimately fall short of the mark. The results of our study underline the need to assess setting-specific needs and concerns when implementing digital technologies. Accordingly, each digitization or digitalization measure, telehealth application, or AI application should be adapted to the respective care setting. Telehealth may have the potential to substantially transform decentralized SOPC; however, despite its availability, it does not appear to be embraced by adult palliative care professionals. The use of a combination of qualitative and quantitative methods has proven to be particularly helpful in this context and may serve as a model. The results provide a foundational basis and can contribute to further research. For this purpose, ethical, legal, and social aspects are of paramount importance, and the perspective of the patient should be incorporated into digital transformation processes in the context of SOPC.

## Supplementary material

10.2196/92048 Multimedia Appendix 1 Supplementary material including the category system for the identified needs.

10.2196/92048 Multimedia Appendix 2 Supplementary material including the category system for the identified concerns.

10.2196/92048 Checklist 1 COREQ checklist.

## Abbreviations

COREQ: Consolidated Criteria for Reporting Qualitative Research
ePRO: electronic patient-reported outcome
HCP: health care professional
mHealth: mobile health
SOPC: specialized outpatient palliative care
TAPE: Telehealth in Outpatient Palliative Care for Adults
