# Supplementary material for: Needs and Concerns Regarding a Pediatric Palliative Telehealth App for Use in Palliative Home Care for Adults Among Providers in Germany: Embedded Mixed Methods Study
Source: JMIR Form Res. 2026 Jul 31;10:e92048. doi: 10.2196/92048 (PMC13427069; doi:10.2196/92048)
Supplement: Multimedia Appendix 1 [file formative-v10-e92048-s001.pdf]

DeepL

Subscribe to DeepL Pro to translate larger documents.
Visit www.DeepL.com/pro for more information.

**Parent category**	**Subcategory**	**Definition**	***Anchor quotes***
**Voice input of content in PalliDoc using voice control**	urban team<br>**Error-free transcription and correction options for voice-dictated content**	Voice input of content in PalliDoc with voice control refers to verbally dictated information that is to be created or executed in the PalliDoc documentation system for various purposes	*Quote 1 (IPA11): "[...] We need a system that learns from speech [...] And for us Westerwald 'basalt heads,' this is something where even the dialect (.) should be more easily transcribed into standard German without errors."*<br>*Quote 2 (IP13): "[...]IP7 just said, in principle, that she (.) um, wants confirmation of what she said/ [...], if [...] there might be errors in it and I don't notice them because I don't have a way to check, [...] that there's a safeguard built in so that when I say something, it reads it back to me. Then I would know/ ."*
	urban team + rural team<br>**Efficient data management and documentation capture via voice input**	Systems and processes for creating and managing tasks to improve work organization via voice input are mentioned.<br>The systematic collection and organization of data as well as the documentation of care-related work processes are described to simplify workflows and ensure data integrity	*urban team*<br>*Quote 1 (IP7): "I'd just say: Hey, make a note right now at 5:05 p.m. that Mr. Müller called with nausea."*<br>*Quote 2 (IPA10): "[...] the automatic entry of extended master data in the basic form. So I [...] admit patients on my own and then have to do all this documentation. [...] it's just very time-consuming."*<br>*Quote 3 (IP1): "[...] if you could also access it via voice control and say: 'Please send the treatment plan to [...] Care Facility XY' or something like that. That, in principle, this can also be triggered directly via voice control and the program is completely interactive, and you can (.) say: 'Um, please send the medication plan there, please call [..] the daughter,' and that this is essentially possible with just a single command."*<br><br>*rural team*<br>*Quote 4: BPC: "[…] we recently had an intern who said it would be great if we could run all our documentation […] through AI […]. And […] I'd find that interesting, because it would actually allow us to spend a lot […] more time with the patient […] and save time elsewhere."*
	urban team<br>**Use of voice control for navigation and information gathering in the PalliDoc app**	includes specifying how the voice input function should be implemented	*(IP2): "I was just thinking, do you need: 'No, continue?' Or wouldn't it also help if the system responded to what you say? That you say what you want to hear?"*
**Voice output of content from PalliDoc using voice control**	urban team<br>**Details on information gathering**	Voice output covers scenarios where content is output via voice output using voice control from the PalliDoc documentation system	*(IP8): "Well, it would save me (..) a significant (.) amount of time—not just for me but also for the patient—if we could just hop in the car at two in the morning. (..) Clicking on the address, that all works fine, and then I say: 'Okay, tell me, Müller, Erich, the diagnoses, last crisis, medications'."*
**Digital capture of patient signature**	urban team **+** rural team	includes the simplification of mandatory signatures on SAPV forms and consent forms	*urban team: (IPV5): "Digital prescription, [...] with a single digital signature [...] from the relative, which is then always valid."*<br><br>*rural team: (IP4): "[...] that we could also have all these [...] slips of paper that we have to get signed, signed digitally. I mean, there are iPads where you can hold all these forms up for them to sign."*
**Introduction of e-prescriptions**	rural team	includes information related to the implementation of the e-<br>prescription function	*(IP2): "The doctors' offices are now, um (..) linked to the pharmacies, and the idea that this might also work for us on-site when we're with the patient (..) is probably not possible (laughs)—that just makes me shaking my head.*
**Scan medication plan to import**	urban team	involves simplifying medication entries in PalliDoc via a digital application	*(IP7): "[...] What I could well imagine, for example, is that you could essentially use a (.) photo—if you have a medication plan—to scan it in via a photo, and that it would then automatically (.) populate the fields for me in PalliDoc."*
**Notification feature**	urban team<br>**Digital identification of crisis patients**	Passages referring to a type of push notification to alert users	*[...] So, just a push notification like that. [...] if you yourself feel that this is information that's important for the person or people on duty after me—whether it's at night, the next day, or on the weekend—so you can just say, "Here's a patient who needs special attention, just like the critical patients we've had before." That you can just pop that up [...].*
	urban team<br>**Digital labeling of the urgency level for medication orders**		*Quote 1: (IP8) [...]The counter (.) would save nursing and administration a tremendous amount of communication and time, [...]*<br>*Quote 2: (IP8): "Medication counter—Pallidoc doesn't have that, for example. No idea why. If I enter [...] Palladon 2 mg, 50 tablets today, when will they all be used up?"*
	urban team<br>**Digital labeling of new entries for prescription creation**		*(IP8): "[...] Well, it would help me if a pop-up appeared, (.) when they enter prescriptions externally.[...]".*
**Verifying the completeness of information via a check function**	rural team	Text passages where a kind of push notification is needed to alert users	*(BA4): "[…] so, a button or […] a little red […] arrow pointing to the physical examination […]. […] with all the documentation, people sometimes get a little lost at the end, I think (laughs).*
**Economic route planning**	rural team	Economic route planning addresses a digital feature that efficiently plans home visit routes for the optimal use of<br>available resources	*(BP1): "[…] what I would find helpful would be […] organizing the route plan or scheduling […] so that you can visually see on a map where the patients are, to make it just a little more economical and then plan the routes (.) […] as scheduling, that would be the optimal route to (..) simply […] the most economical."*
**Data availability ePA**	rural team	Data availability refers to passages that address the need for data on medical history and treatment history to be available for a complete palliative care assessment	*(BP3): "It's not just about doctors' notes. It's also about power of attorney for healthcare and living wills. Then you're constantly calling the hospital, they don't have the paperwork, you don't know who the contact person is. And it's very tedious [...] to collect this data, which is, however, (..) essential for us to be able to provide care (...). Yeah, it would be kind of cool if there were […] a digital patient record—one that's secure in terms of data protection, of course—but where healthcare providers could access it temporarily via encryption to get to that data (...)."*
**Teleconsultation**	rural team	Teleconsultation includes all text passages that mention teleconsultation	*Quote 1: (BP2): "Especially on call, I think it's really good when, um (..) you don't have a doctor on site right now or a colleague. And that the doctor doesn't have to come and you can connect with them (..) maybe in a critical situation, and then (..) that person can stay at home and I can handle it on site, in the acute situation.*<br><br>*Quote 2. (BZ2): "[…] as we both just mentioned, because, um, what we'd like to eliminate or change or something like that are the many meetings. We have an awful lot of meetings all the time and everywhere […] but I definitely see an opportunity in some of those meetings, when they're held digitally, to save time […].*
**AI-generated content**	urban team	describes that AI can only select summary content and make it available for input and output<br>[…]	*(IPA 11): "It goes so far that I could then say, (.) when I have conversations with the patient or their relatives, that I could then provide that in keywords (..) as a task. (.) Or, even better, of course, would be if the program recorded what I (.) discussed with them and then just gave me keywords (..)".*
