# Supplementary material for: Needs and Concerns Regarding a Pediatric Palliative Telehealth App for Use in Palliative Home Care for Adults Among Providers in Germany: Embedded Mixed Methods Study
Source: JMIR Form Res. 2026 Jul 31;10:e92048. doi: 10.2196/92048 (PMC13427069; doi:10.2196/92048)
Supplement: Multimedia Appendix 2 [file formative-v10-e92048-s002.pdf]

Subscribe to DeepL Pro to translate larger documents.
Visit www.DeepL.com/pro for more information.

Parent category	Subcategory	Definition	Key quotes
**1. Lack of trust in AI-generated content**		Lack of trust in AI encompasses concerns regarding blind faith in automation and the delegation of responsibility for assigning certain facts to the documentation system primarily via technology-based speech recognition<br>(FG1 BA2): "[...] That means I would probably check everything and possibly spend just as much time on it as if I were typing it in quickly myself.	(FG1 BA2): "[...] That means I would probably check everything and possibly spend just as much time on it as if I were typing it in quickly myself. Because especially when you use AI (.) [...]."
			(FG2 IPA 12): "That I wouldn't have that much trust in the AI, um, that it would summarize it for me like that, (..) that the accuracy of the summary is correct."
**2. Uncertainty regarding legal certainty, data protection, and privacy when using telehealth**		Includes the legal, security-related, and ethical challenges in handling personal data due to the use of digital applications	(FG1 BP1): "[...] But I'm also a skeptic, um, an opponent of the digital medical record [...] Because I think that some diagnoses aren't important for an ENT doctor. And there's also quite a lot of, um, yes, prejudgment, (.) when you see various things [...] I don't think that [...] is good. [...]."
			(FG2 IP7): "I just said that then every patient would have to sign the same thing we just signed if conversations . You certainly can't just do that for data protection reasons."
			(FG2 IP3): "Although I don't actually know right now whether that (.) is allowed in terms of traffic regulations, right? (.) That's probably going to be the bigger problem, because 're supposed to look at the road while driving, not while driving/"
**3. Negative Effects of Delegating Responsibility to AI**	**3.1 Loss of staff competence due to AI**	Changes and effects that may negatively impact the SAPV team due to the use of telehealth	(FG2 IP7): "Well, one concern might be, um (.) that we rely too much on this system, (.) in a certain way, right? That you just dictate something there or have something read back to you and no longer (..) um, read it over again [...]."
	**3.2 Job Security Threats Due to the Use of AI**		(FG1 BP5): "I'm a little worried that I'll lose my job (laughs) ((laughter)). Yeah."
	**3.3 Lack of clinical skills**		(FG2 IP7): "[...] I'm standing with the patient and am supposed to do an ultrasound [...] (.) or listen to a patient's chest. I never learned that during my training (.) and I would feel, [...] even if I, um, have a doctor on the other end of the phone, [...] well, a bit overwhelmed [...]"
	**3.4 Overwhelm from multitasking**		(FG1 BP1): BP1: "I'm generally a bit critical of this, especially in the car/[...], the hands-free system is often very loud, and when I'm stopped at a traffic light/[...], even if we don't have names on the screen, I find that a bit problematic. And sometimes we have so much on our minds, and then I have to concentrate on what's being said again. The only thing missing is me eating my breakfast roll at the same time. I think you have to differentiate a bit #20:52:00#
**4. Lack of technical feasibility**	**4.1 Lack of improvement through e-prescriptions**	The integration of digital applications into the SAPV is accompanied by practical and infrastructural challenges or obstacles	(FG2 IPA12): "Yeah, I know what I mean, (...) because it doesn't work."
	**4.2 Concerns about the availability of technical resources and infrastructure for teleconsultation**		(FG1 BP3): "[...] But it also depends on a good network, enough battery life, enough data, and patient consent."
	**4.3 Verbatim voice input produces texts that are too long**		(FG1 BA4): "[...] for the body text, [...] that doesn't make much sense to me either. [...] when you then [...] dictate it in, it's such a huge amount and so unnecessarily long, [...] no one reads that anymore because there's so much rambling, [...] I think."
	**4.4 Lack of training**		(FG1 BP6): "It just has to be taught well. I'm really a total klutz when it comes to stuff like that. I'm not very good at any of it, but I think there needs to be good training."
**5. Negative social impacts of telehealth**	**5.1 Effect of distancing**	Includes personal, emotional, and subjective limitations that affect the nature of relationships and care provided to patients and family members in the context of a digital application	(FG2 IPA6): "[...] And for me, the conversation is important. I drive to the patient's home and talk to them [...] And if I need to add something later, I look at my notes (.) and enter it afterward, not on the patient or anything like that. (..)."
			(FG2 IPA 11): "Because the most important thing for me (..) is missing in all video conferences. (...) Body language"
	**5.2 Lack of in-person support**		(FG1 BP4)[...]Just imagine, someone calls me at 2:00 in the morning. A wife, totally overwhelmed. [...] And then I say: "Yes, please turn on the camera," and I feel really ridiculous.[...] So, I also think that a bit of the human touch gets .
			(FG2 IP7) "[...] Also, more out of concern that, um, personal contact is [...] minimized as a result. [...] it might also tempt you to, well, um, skip a home visit in person and just do a tele-consultation [...] So the scope of application would have to be very narrow, so that it's really only used for emergency situations and only for patients who are actually able .
	**5.3 Lack of professional boundaries due to contact with patients via messaging services**		(FG1 BZ1): "But then it got so out of hand, um, that I, um, just... received a text message almost 24 hours a day, um, sometimes even in the middle of the night [...] me, it was difficult to re-establish that boundary [...]."
**Residual category: General concerns regarding telehealth**		Digital communication can be unhelpful when it comes to assessing subjective impressions	(FG2 IPA 11): "[...], but that [...] interpersonal conversation on the phone—that's what (.) actually requires feedback/ (..) in my view. (..) An app won't help us there either (.) (speaking softly), because the individual (.) subjective impressions, (..) which I can only get during a phone call, have several levels.[...]
